# The Incalculability of the Generated Text

**DOI:** 10.1007/s13347-024-00708-0

**Published:** 2024-02-17

**Authors:** Alžbeta Kuchtová

**Affiliations:** Institute of Philosophy, VVI, Slovak Academy of Sciences, Klemensova 19, P.O. Box 3364, 813 64 Bratislava, Slovakia

**Keywords:** Machine learning, Machine creation, Generative literature, Derrida, Iterability

## Abstract

In this paper, I explore Derrida’s concept of exteriorization in relation to texts generated by machine learning. I first discuss Heidegger’s view of machine creation and then present Derrida’s criticism of Heidegger. I explain the concept of iterability, which is the central notion on which Derrida’s criticism is based. The thesis defended in the paper is that Derrida’s account of iterability provides a helpful framework for understanding the phenomenon of machine learning–generated literature. His account of textuality highlights the incalculability and mechanical elements characteristic of all texts, including machine-generated texts. By applying Derrida’s concept to the phenomenon of machine creation, we can deconstruct the distinction between human and non-human creation. As I propose in the conclusion to this paper, this provides a basis on which to consider potential positive uses of machine learning.

## Introduction^1^

The French philosopher Jacques Derrida wrote extensively on technology. In this paper, I argue that his theory of exteriorization can be applied to the difficult question of the relation between machine creation and human creation. In what follows, I focus specifically on textual creation. I explain what I mean by textuality in the section on iterability, relying specifically on Derrida’s non-anthropocentric definition of textuality, which includes any material or non-material inscription that has meaning. This definition of iterability erases the distinction between speech and writing, as both are understood as based on iterability. This deconstruction of the difference between speech and text is one of Derrida’s best-known tenets, and thus I build on it as a presupposition that is widely accepted in literary studies (Derrida, 1998: 10–26). According to Bernard Stiegler, recent developments in artificial intelligence can be viewed as the latest stage in the exteriorization of the imagination (Wellner, 2021: 200). Both Stiegler and Derrida describe technologies as *pharmaka*, a concept that, as its original Greek meaning suggests, denotes either a “remedy” or a “poison,” depending on how it is used (Pavanini, 2022: 11; Vlieghe, 2014: 529). As we will see, I agree that artificial intelligence (AI) is a *pharmakon* and thus that it can have both positive and negative effects depending on how it is used. While distinctions such as “positive” and “negative,” “good” and “bad,” lose all meaning, my aim is to direct us toward the possibility of imagining the remedial effects of AI rather than focusing on its poisonous consequences (I will refer to these as “positive” only for the sake of simplicity, fully acknowledging that the concept of a *pharmakon* erases the distinction between good and bad). As I will illustrate, some philosophers view our current uses of large language models (LLMs) as a poison rather than a remedy. Stiegler is both critical of and receptive to contemporary technologies: he is critical of AI because he relates it to the capitalization of the imagination, which renders it computational and automated (2017a: 79–99). Indeed, he claims that “understanding (*Verstand*) has been automatized as the analytical power delegated to algorithms executed through sensors and actuators operating according to formalized instructions that lie outside any intuition in the Kantian sense—that is, outside any experience” (2017b: 27). According to both Stiegler and Galit Wellner, creativity as such presupposes embodiment and imagination, and thus AI is devoid of true creativity, incapable of authentic textual production. Other authors, by contrast, claim that AI can involve affectivity and embodiment (see Parisi, 2019). Others still, echoing Heidegger, fear that machines will one day replace humans. Some of these authors, such as Sean Dorrance Kelly, deny that machinal creativity has the status of authentic creativity, precisely because it precludes singularity: “such a machine would not be evidence of the singularity; it would not so outstrip us in creativity that we couldn’t even understand what it was doing” (2019). This fear is likely also linked to the fact that certain machine-created artworks can pass the Turing test (and are thus indistinguishable from human creations), as Mark Coeckelbergh observes (2016: 288). Some have argued that machinal creativity is not authentic creativity insofar as it lacks a genuine origin (Stiegler, 2017b: 290). The term “stochastic parrot” (Arkoudas, 2023) has been used to refer to the LLMs on which ChatGPT and other tools are based, implying that LLMs merely copy data provided by humans and cannot generate original outcomes. This touches on the question of the originality of artworks, including literary works produced by LLMs.

A cluster of questions thus form the background of this paper: What is the basis of our concept of the “essence” of literature? Is this supposed essence tied to originality or unrepeatability? What is the relation between originality and unrepeatability? Are these identical concepts? In what follows, I claim that the originality that is closely tied to our concept of the essence of literature and of the living present is distinct from unrepeatability. This paper is not primarily concerned with whether, or to what extent, LLM-generated texts are deterministic (predictably repetitive) or stochastic (unpredictably creative), although it does touch on this very interesting question. Rather, it focuses on how to treat the *products* of different kinds of machine-like mechanisms—more concretely, the literary texts LLMs produce—and on their reception in philosophy and literary studies. Are they to be considered literature on a par with “purely human” works? I claim that Heideggerian and metaphysical presuppositions continue to underlie the contemporary discourse on literature and creation. My conclusion is that there is no such thing as *purely human* literature and that being the outcome of human creativity does not belong to the essence of literature. Rather, as Derrida recognizes, the concept according to which we should define literature is not “originality” but rather the “secret.”

As I will show by drawing on Derrida’s account of textuality, while it is true that the ongoing capitalization of data has radically increased “the hegemonic power of Google, Apple, Facebook and Amazon” (Stiegler, 2017b: 25), this capitalization is not necessarily metaphysical. Stiegler claims that objects created under the conditions of fully computational capitalism are not infinitizable (2017b: 23). The capitalization of algorithms and of data is indeed a real problem, as Luciana Parisi argues (2015), but it is not essential to the functioning of AI. Rather, it is the result of malfunctioning political systems, including democratic systems, which allow the exploitation of AI. Parisi argues that this is linked to a refusal to recognize the incalculable at the heart of algorithmic mechanisms, itself rooted in a techno-capitalist logic according to which everything must be calculated and solved:

Far from being liberating, the deposing of inferential reasoning is constantly advertised to us as the ability of networked capital to package social complexity in profiles available to us at the touch of a button. Within this context, the real challenge today is perhaps not to map human–machine–animal non-conscious cognition, but to critically re-address the function of reason and to theorize—rather than reject—the automated use of inferential reasoning as part of a general artificial thinking. (Parisi, 2019: 104)

In response to these ideas, I will show that there is something that resists capitalization even in the case of generative literature. My inspiration for this interpretation of AI is Derrida’s account of technology, in particular his concept of iterability. Derrida is also critical of technologies, however, and he recognizes that the digitalization of communication raises difficult questions about user privacy (Sjöstrand, 2021: 145–149). As he observes, personal information has become part of a digital archive that is potentially open to breaches. In addition, he argues that the problematic of the *actualité* and the speed of information should be critically rethought (Derrida, 2002: 29–31).

In the following sections, I focus on generative literature rather than ethical problems connected to the possible uses of AI. “Generative literature” is a term that refers to literature generated by machine learning. Examples of such literature include texts of various kinds generated by LLMs such as the neural network GPT-2, including a small number of published collections of generative poetry. Thus far, only one such collection has been published under the name of the neural network that created it (the 2020 collection *Výsledky vzniku* by Liza Gennart (Gennart, 2020), a GPT-2 neural network).^2^ This particular literary use of neural networks, in which authorship is assigned to the network itself, is quite new; up to now, neural networks have largely been used to manage social media, internet shopping, customer service, and research. The literature on such technologies therefore often focuses on ethical issues regarding safety, responsibility, and trust in the context of social media (see Bezuidenhout & Ratti, 2021; Boem & Galletti, 2021; Ratti & Stapleford, 2021; Glavaničová & Pascucci, 2021, 2022).

In this paper, I focus on generative literature, which bears certain similarities to another type of literature that has existed since the 1990s: digital or electronic literature (i.e. literature that takes the form of a digital medium, code, or, as Hayles puts it, is “digital born” (Hayles, 2008). The problem that is my focus here is thus grounded in a long tradition in the literature. Concepts such as “cybertext,” “technotext,” “software studies,” and “critical code studies” have been present in the literature for some time (Husárová, 2015). Examples also include automatic translations that can be considered generative creations even though they differ from literature in important respects. I will mention such works only briefly in this paper insofar as examining them more closely would require elaborating on the much larger issue of the difference between generative translation and generative literature. With that said, I believe that the account offered here can be applied to translations as well.^3^

From a phenomenological point of view, I will inquire into whether the Heideggerian concept of *ποίησις*, defined in terms of the manual (mostly human) production (creation) of *άλήθεια*, can be applied to this case. I will argue that Derrida’s critique of Heidegger’s understanding of the concept of production (creation) sheds light on the positive aspects of the machinal creation involved in generative literature and machine learning translations. My claim is that Derrida’s theoretical framework of exteriorization is applicable to recent developments in machine learning and is useful for explaining it. This is because Derrida presupposes that what he calls the “secret” (the incalculable, the infinitizable, the incomputable) exists in all texts—including, as I will claim, generative texts. My conclusion is that the Derridean condition of creation—the secret, the incalculable—can serve as a starting point for theoretically explaining new uses of LLMs (for example in generative poetry). The novelty of this approach consists in using Derrida’s concept of the incalculable to analyze machine creation, since the incalculable, as an element that is inherent in literature, has yet to be discussed in the literature on computational creation.

Derrida’s theory of exteriorization may offer a theoretical solution to the problem of the capitalization of information, as formulated by Stiegler. As I will show, Derrida formulates the same question in slightly different terms. His account also provides a useful perspective from which to contemplate not only negative but also positive uses of machine learning—uses that go beyond strictly capitalist ones (e.g. in cars, for research, and to facilitate shopping and communication with customers) and the hegemony of the economic imperative. In my conclusion, I propose concrete examples of possible uses of machine learning that fall firmly on the “remedy” side of the remedy/poison distinction.

In this paper, I will rely on the Derridean concept of exteriorization. Exteriorization in writing is a process of becoming external to human consciousness. Derrida deconstructs the opposition between exteriority and interiority by appealing to the example of writing. He thereby also deconstructs the dependence of writing on human consciousness and speech, which in the history of Western philosophy has been considered absolutely interior. Unlike speech, writing has traditionally been considered secondary precisely because it is thought to be external to human consciousness (interiority). By contrast, the spoken word is viewed as internal due to its immediate proximity to consciousness—due to the lack of intermediary between the two—and thus as proper to the interiority of consciousness. On this view, the written word is “externalized” through the use of a third element (paper, a pen) to transfer meaning. As Björn Sjöstrand observes, writing, for Derrida, “functions as a technological prosthesis, an *externalization* of memory, whose contents can be preserved in a way that is unquestionably objective and passed down to future generations” (Sjöstrand, 2021: 56). In short, Derrida claims that all words have a technical element and that everything that exists is already, in part, also technical and algorithm-like (while also being, again in part, ungraspable, secret). In the history of philosophy, the written word has not been considered authentic because it is thought not to relate immediately to interiority (and thus not to be proper to it). Therefore, Derrida argues that the written word has traditionally been viewed as a supplement for the spoken word, and thus as inferior. In *Of Grammatology*, Derrida deconstructs this hierarchy by appealing to the concept of iterability, on which both the spoken word and writing are based. He shows that Heidegger used precisely this type of argument, based on the notion of proximity, to support his distinction between handwriting and mechanical writing (typewriting). Similar concerns have been voiced by more recent thinkers, grounded in the same assumptions regarding proximity, authenticity, and the relationship between exteriority and interiority. Of particular concern in contemporary discourse is *authenticity*: authentic writing is thought to have been composed by the human mind, making generative writing inauthentic. The human mind is still considered a space of authentic interiority that is proper to human beings alone. Human writing is conceived of as proximate to the “soul,” the mind, or the living present. What has long gone unquestioned, however, is the presupposition of the uniqueness of the human soul, which is itself based on a Christian (Heideggerian) metaphysics that views the soul as having been made in the image of the divine. In the following, I will draw on Derrida’s thinking to deconstruct the oppositions at the heart of this conception, and thus the opposition between human and non-human writing.

## Heidegger and Technique

Heidegger’s definition of technique rules out the possibility of machines engaging in autonomous creativity equivalent to human creation. Heidegger’s work betrays a certain phobia of machines, even though he claims that the creation/production of άλήθεια and of being is technical. He calls this production of άλήθεια *poiêsis*. However, production is not machinal for Heidegger; it is linked to technique, not technology, which he distinguishes (Heidegger, 1968: 13). He defends the importance of manual work and of the human hand, claiming that it is only the hand that can authentically unveil the truth and engage in *poiêsis*, which he defines as the creation of truth, but also of art, for example poetry.^4^ For Heidegger, manual work is a species of thinking that does not involve the use of supplements, and thus exteriorization. This is because the hand has a special, immediate connection to being insofar as it is a living present. Heidegger associates the hand with the word to the extent that both belong to the essence of being. His conclusion is that the only authentic form of writing is handwriting.

Derrida criticizes this thesis and associates the relation between the hand and speech with the problem of writing and handwriting. His conclusion is that in handwriting there is a relation to exteriorization because iterability and technique are in play (I will explain this further below). In *Heidegger’s Hand,* Derrida detects a link between Heidegger’s nationalism and this preference for “traditional” modes of production and his phobia of machines.

According to Heidegger, there is a danger that the traditional and conservative production of truth will be disturbed by modern techniques that are perversions, on his view, insofar as they endanger human autonomy (more precisely, the autonomy of the hand) (Heidegger, 1992: 50). The only pure thinking, for Heidegger, is the thinking that is done by hands. Heidegger writes that the hands speak the most purely when the human being works with them silently—or when the human speaks, as speech is also the source of originality, as part of the living present. This is how the human being gains access to the essence of being and truth (άλήθεια). The essence of technique must be separated from machinality. The machine mutates and changes our relation to the essence of being; it is understood by Heidegger as external, interrupting the immediate relation of the human hand to being—a relation that is formed by touching the piece of wood, for example, but also in poetry. This is because the essence of technique is *ποίησις* (handiwork), which is the origin of creation and of the movement of truth. Heidegger suggests that this essence should be protected and thus that we should return to manual creation. He was critical of even the typewriter as a means of creative production; on his view, a good poet writes with his hands, not on a keyboard. Although few philosophers today would critique the use of computers in writing, many remain critical of AI, precisely because they rely on the same presuppositions as Heidegger, in particular his assumptions regarding authenticity, essence, being, human, and the living present. Not only does this framework secure the privileged position of the hand, or of purely human creation, but it also entails a phallocentric definition of technique grounded in a phobia of machines and new technologies.

We should therefore expand Heidegger’s definition of technique, extending it beyond human creation to encompass phenomena such as generative and digital literature, which are now considered authentic literature by many, on a par with texts produced by more traditional means. Heidegger would have viewed generative literature as perverted—as depriving humans of their privileged place in the universe. Precisely because of the anthropocentric assumptions underlying his view, however, his approach ought to be rejected.

## Iterability

The concept of *iterability* was coined by Derrida, who borrowed it from Husserl and gave it a different meaning. Derrida claims that life and everything that is contains an external, technical element (Naas, 2012: 202–227). This means that everything is iterable, repeatable, codable, or algorithm-like. Derrida defines iterability in terms of the possibility of being repeated or coded. His analysis is based on the concept of *différance*—a concept that stands for the abstract origin of everything that exists but which at the same time implies that everything is differentiated and therefore iterable and coded, computable and exteriorized. *Différance* entails that there is no origin at all, and thus no pure, uncomplicated origin of writing, no purely human subjectivity in which meaning originates. As Derrida writes, “[h]aving no essence, introducing difference as the condition for the presence of essence, opening up the possibility of the double, the copy, the imitation, the simulacrum—the game and the graphe are constantly disappearing as they go along” (1981: 157). By this he means that there is no essential “authentic” meaning from which secondary meaning is derived, as is sometimes thought to be the case for the meaning generated by LLMs, which is viewed by many as a copy, an imitation, a simulacrum, a “doubling” that is not to be taken seriously. Derrida claims that machine-like mechanisms are considered “doubles” of the human, mere copies to be erased, perfectly controlled, or even enslaved. Hence the rise of the term “stochastic parrots” to refer to LLMs, the implication being that they merely copy and paste human material without having any real understanding of its meaning, just as a parrot lacks any understanding of what it is saying when it mimics human speech (see Arkoudas, 2023: 1–29). The metaphor of the parrot as a mimic that lacks any understanding of what it is saying is certainly problematic in itself, based on a Heideggerian hierarchy between human, animal, and thing (see Derrida, 2008: 155–156). This hierarchy is again grounded in the concept of *authenticity.* Within the framework of this metaphor, LLMs are like the figure of the marionette in Paul Valéry’s *Monsieur Teste*, mere doubles of humans who must be killed simply because they are not authentic and derive from human beings, who represent their origin. In Valéry’s novel, Monsieur Teste views himself as a kind of marionette, a machine-like thing, and is convinced that he has a double inside of him (that *différance* resides within him). According to Derrida, however, the human who seeks to kill the machinal element in himself is already a marionette, a machine. This metaphorically illustrates the similarities between Valéry’s and Heidegger’s conceptions of the problem of authenticity and provides a clue as to how the Western discourse on human essence might be deconstructed:

But if the narrator speaks of the marionette that kills this other marionette that Monsieur Teste is as a fictional character, this same narrator is already himself a sort of marionette, both because he is manipulated and ventriloquized, as a theatrical fictional character by Valéry, and because he identifies himself without delay with this other marionette, Monsieur Teste, just where Monsieur Teste claims to have killed the marionette within him. (Derrida, 2009: 188-189)

Monsieur Teste’s behavior is automatic, and he has a double who causes his narrative to be differed (*différé*), who writes automatically as the narrator of his own story—as a writer. He wages a war between these two identities, from which the machine-like identity is to be erased. Yet according to Derrida, we cannot kill the double, the other, the machine-like identity that takes the shape of a writer (either inside or outside of us) precisely because it is a central part of *différance,* of writing as such. The possibility of being imitated—of being copied, of becoming double—is always there, and this is why we are able to express ourselves; it does not signify our end or stand before us as a pure threat.

What does the possibility of always being copied entail? When Derrida writes that “[t]here is nothing outside of the text” (1998: 158),he denies the existence of the non-iterable. There is only the sphere of *différance,* where everything is different from the origin and possibly copied, iterated*.* What he means is that there is nothing that cannot be differed, doubled, repeated, iterated, or coded. The word, speech, the text, is here a synonym for the doubling mechanism, the *différance*, the repeatable, the codable, the iterable. Iterability means that “[t]he possibility of repeating, and therefore of identifying, marks is implied in every code, making of it a communicable, transmittable, decipherable grid that is iterable for a third party, and then for any possible user in general” (Derrida, 1982: 315).

Indeed, according to Derrida, nothing is such that it cannot be iterated in any system. Without iteration and *différance*, there is no meaning. The absolutely non-iterable is impossible, a pure nonsense. A meaning that we cannot repeat does not mean anything at all. Therefore, there is no such thing as an immediate (authentic) relation to the interiority of being, the soul, or consciousness—one that does not involve the intermediary of the hand or of the spoken word. The hierarchy between interior (original) and exterior (supplement or secondary) cannot be maintained, as Heidegger had hoped. Both interiority and exteriority are based on iterability. Speech, handwriting, and typewriting, and indeed any other machinal type of writing or any other way of communicating meaning (including writing performed by an LLM), are all external to the interiority of consciousness. Indeed, Derrida would go even further, suggesting that even the interiority of consciousness is external to itself. On his view, there is no pure interiority; there is only *différance*, which could potentially be considered the source of authenticity (although in truth *différance* implies that the concept of authenticity no longer makes sense). With that said, we should be careful not to reduce the concept of *différance* to technology; as Roberts (2005), Bennington (1996), and Sjöstrand (2021) show, these are different concepts. As these authors argue, the concept of *différance* is an abstract notion that cannot be reduced to material technology, *pace* Stiegler. *Différance* is based on iterability; it is the source of the performativity of writing. In LLMs, writing becomes “other” to itself, its own “double.” *Différance* thus makes both generative and human writing possible.

## The Paradox of Calculability

In *Papier Machine* (2001), Derrida admits that certain unconscious, non-human, non-affective events can have an esthetic impact on the living (for example on humans). In this text, Derrida imagines the machinal production of an event (a literary work, for example, although the term “event” could apply to any artwork), whose mechanical production was itself programmed by a machine.^5^ We can apply his theory to the contemporary example of texts generated by LLMs that are themselves managed by supercomputers. Derrida acknowledges that such a scenario involves a paradox between the event (representing singularity) and the machine (an automatic device that produces something through repetition). In what follows, I will present his formulation of Stiegler’s concern about the capitalization, or the calculability, of the text, as mentioned in the introduction. I will then focus on explaining how this paradox arises.

Derrida’s analysis of Rousseau’s texts in *Typewriter Ribbon: Limited Ink (2)* shows that Rousseau’s writings contain an unconscious element, as if his oeuvre were independent of its author.^6^ As Derrida writes:

The work will accomplish its work of work, son oeuvre d’oeuvre, beyond its signatory and without his living assistance, whatever may be the time required, whatever may be the time to come; for time itself no longer counts in the survival of this “sooner or later.” The time that this will take matters little, time is given, it is on my side, it is taken and has taken sides in advance, thus it no longer exists. Time no longer costs anything. Since it no longer costs anything, it is graciously given in exchange for the labor of the work that operates all by itself, in a quasi-machinelike fashion, virtually, and thus without the author’s work: as if, contrary to what is commonly thought, there were a secret affinity between grace and the machine, between the heart and the automatism of the marionette, as if the excusing machine as writing machine and machine for establishing innocence worked all by itself. (Derrida, 2002: 86-87)

Derrida shows that even texts written by a human being, in this case Rousseau, are the result of certain automatic mechanisms that are independent of the human being. In this analysis of Rousseau’s texts, he affirms the existence of something like “Rousseau’s machine” (2001: 87), arguing that his texts involve their own type of automatism. Indeed, Rousseau himself uses the expression “machinelike effect,” but Derrida claims that there are many other examples of machinal operations in Rousseau’s autobiographical texts. Rousseau’s autobiographical texts repeat themselves and thus exhibit a pattern (for example the pattern of repeated excuses and the repeated begging of pardon).

In *Typewriter Ribbon: Limited Ink (2)*, Derrida identifies the mechanisms of the written autobiographical genre that he calls “confessional writing.” In his analysis of confessional writing, which here serves as an example, Derrida mentions the mechanism of a confession that functions independently of the confessing author. Such confessions can be exteriorized from consciousness, from the soul (the living present), and can take the written form, as is the case in Rousseau’s and Augustin’s writings. It can then be produced by an algorithm as well. Derrida describes this machinal effect of exteriorization as leading to an infinite repetition of words of regret. Because the written demand to be excused, formulated as a confession, exacerbates the author’s guilt, the excuse must be repeated. The very inscription of the confession puts the textual machine (a term that Derrida borrows from Paul de Man) to work. This is how the repetition of the textual event occurs. Derrida emphasizes the erasure of the *I* in the text (2001: 99). This means that, with its possible future impact, the text can work on its own, even after the death of the author (2001: 103). Adding to this, Derrida also imagines the main characters involved in this confessional narrative and the infinite repetition of their lives (of the living present) in a virtual library.

Derrida does not distinguish between material and virtual exteriorization, because in both cases the writing is partly an automatic mechanism. The handwritten archive is already artificial and based on machinal mechanisms. Every trace, defined as an exteriorization of consciousness, is always already artificial. Any archived document is transformable, alterable—including Rousseau’s original manuscript (Derrida, 2001: 145). Rousseau’s and Augustin’s writings are guided by the “first machine,” which is tied to the unconscious mechanism of the writing itself. This applies to all types of text, whether on screen or on paper. The calculation of the motives of the text is defined in advance, without the author’s knowledge, because it is guided by the automatic mechanism of the first machine. As Derrida writes: “This is a first machine, the implacable and repetitive law of an undeniable program; this is the economy of a calculation inscribed in advance” (2001: 104). In this passage, the “economy” that guides the writing is a reference to the unconscious mechanisms that are in play, but also to the infinite chain of meanings that words can have (one word referring to another, and so on), as well as to the mechanisms (laws) that govern the use of idioms and grammar.

Derrida’s concrete analysis of confessional writing (Augustin, Rousseau) takes into account the trauma of the desire of the living movement that belongs to the body implied in the confession (the living present). This body is injured and threatened by the machine’s work, which results in expropriation. Thus the virtualization of the event by the machine exceeds the classical philosophical opposition of the possible and the impossible. Hence the paradox mentioned above, which consists in the fact that while the machinal principle of repetition makes the production of the event possible, this repetition “traumatizes” the singularity of the event, as the latter is situated in the living body of the confessing person. More concretely, in the case of confession, the repeated excuses do not have value and cannot be determined in advance by the law of iterability. The begging of pardon and the excuse are original, unrepeatable, singular events, and are thus secretive. Calculation (marked by repetition) therefore makes confession both impossible and possible. This is a version of the Derridean deconstruction of the Christian concepts of consciousness and authenticity, applied to confession and writing. Put differently, the claim is that originality is impossible in confessional discourse because the latter repeats itself in various ways. According to Derrida, this analysis can be applied to all texts, not only autobiographical confessionals.

## Conclusion

When it comes to coded text, or to texts produced by machine learning, Stiegler claims that the text is “always identically repeatable” (2017b: 212). By this he means that in the process of repetition no new elements are involved; the text is absolutely repeatable. If this were true, coded texts could not be considered autonomous creations. Stiegler states that our current stage in the exosomatization of the noetic faculties in general is based on information, data which “can only present itself as *formatted in terms of its apriori calculability*” (2017b: 81). In saying this, Stiegler is pointing out that AI and the exteriorization of knowledge based on information can only be based on pure calculability, pure predictability, where nothing new or secret enters into the process. This is because AI is based on pure homogeneity. According to Stiegler, the element of the non-calculable is utterly absent; there is nothing that exceeds comprehension (*Verständnis*) or understanding (*Verstand*), because there is no event that is linked only to embodied experience. As he specifies in *Technics and Time 2*, information (as an element with which AI works) is not unrepeatable *in principle*, because “its repetition is an exhaustion of its value” (2009: 137). In *Automatic Society vol. 1*, Stiegler claims that this type of exteriorization is not subsequently internalized (it need not be located in cerebral structures) and is based on tertiary retention (digital memory). He also criticizes the informatization of knowledge in the form of digital archives and does not see how they could be of use to capitalism, because they are not interesting from the standpoint of the market. He further claims that digital memory promotes de-territorialization and, rather than supporting the formation of a political community, instead de-composes communities. By contrast, material inscriptions in the form of writing, as a kind of material exteriorization, create political communities.

Derrida has a response to this problem, however; he claims that the codable necessarily also entails the incomprehensible, the secret. If this is true, then texts do not lose their incalculability, even when they are created on the basis of computability and on the principle of mimesis. As Derrida writes in the preface to *Dissemination*, no concept escapes deconstruction. Deconstruction involves a double movement: first, all concepts are situated within a conceptual system; at the same time, it is true that any one concept is exterior to that conceptual system. Therefore, there is nothing like a fixed system of concepts that is to be deconstructed. Instability is interior to the system and to information, as is codability. It follows from this that writing is never completely finite or defined. In any kind of writing, there is a movement toward exteriority, toward *différance*.

As a consequence, even if everything can be coded or programmed, this does not mean that (virtual or material) reality does not include things that cannot be calculated. Thus even texts that are purely based on calculability and on code (generated by LLMs) contain an element of incalculability, just as texts produced by a living author do. With that said, no text can be “purely” based on coding mechanisms, because nothing is purely iterable, according to Derrida—just as there is nothing that is purely singular or original, in the sense of being absolutely unrepeatable (authentic).

In an interview with Stiegler in *Echographies of Television*, Derrida claims that the reappropriation of exteriorized meaning can never be absolute, precisely because of iterability. If it were absolute, there would be no sense. Even so, the reappropriation of meaning is important. Derrida identifies the paradoxical relation between *appropriation* and *reappropriation*—in other words, between the “proper” and the “other.” As he observes, “there is meaning only insofar as this process of appropriation is, in advance, held in check or threatened by failure, virtually forbidden, limited, finite: meaning does not depend on me, it is what I will never be able to reappropriate totally” (Derrida & Stiegler, 2002: 111). He calls this double movement *ex*-*appropriation*, claiming that if one were to appropriate meaning perfectly through machine-like mechanisms, there would be no otherness, including otherness in the form of exteriorized meaning (written, coded meaning).

Derrida therefore shows not only that everything is codable (iterable) and that mimesis is therefore omnipresent, even in creation, but that the process of mimesis is imperfect, that the process of transmission is always disrupted. This is also true of writing generated by LLMs, for on Derrida’s view all writing, even handwriting, is machinal and automatic: the hand is also a tool, as are the voice, the keyboard, and the pen. Both LLMs and human thinking are coded processes, because both are iterable. Derrida claims that the hand, speech, and programming tools are all means of exteriorization, and none is “worse” than the other per se. On the contrary, he claims that just as the absolute secret does not exist, just as nothing exists outside of the text, absolute codability does not exist. It follows from this that even a coded text created by machines is not absolutely repeatable: none of its elements can be repeated such that it is always absolutely identical to itself.

Put simply, this means that any element that is repeated is always necessarily repeated in the different contexts in which it is deciphered, read, or otherwise received. The meaning of the element depends on this context; it cannot be separated from it. We might say that Derrida’s definition of meaning is contextual and performative, as opposed to Platonic. We might even compare it to Wittgenstein’s definition of meaning as use. Consider, for example, the sentence “I like you.” We cannot determine its meaning in itself because this meaning is always changing according to the context in which the sentence appears, even if, from a graphic point of view, it is repeated identically. In each concept, we find movement towards an infinite chain of meanings that leads to ungraspability, to the secret. Derrida refers to this process of enabling the secret as “dissemination.” The secret is definitive of all writing, both human and post-human, since all writing works with concepts that are continuously falling apart. For Derrida, the secret is always already incorporated in any straightforward transmission of meaning, which means that it is internal to the creation of meaning, whether human or post-human. He claims that every sentence is materialized (spoken aloud, written, emitted) in a different setting by default. The world, like everything that is, is always changing, and every situation in which someone or something receives meaning is unique by definition. This is trivially true. Therefore, no element of meaning can be absolutely identical in every context, because its identity is defined by the context, which is always necessarily different (Derrida, 1988). Each element of the text is connected to the web of meaning to which it refers, and what it refers to cannot be decided or determined, for in every context the set of meanings is changing. This aligns with Derrida’s claim that everything that happens (as an event) is both a repetition and a singularity. Thus the event in itself (what is happening, but also the artwork, writing) is aporetic and paradoxical.

Derrida’s concept of meaning provides a helpful framework for understanding how LLMs function. Hannes Bajohr observes that an LLM can learn to process data in such a way that it learns its inherent patterns, structures, and variations. It is then able to generate new data in a way that is strikingly similar to human production. We might say that it is able to recreate something that is the “same” as something new. It is “representation as repetition in a different mode” (Bajohr, 2023), which is precisely how many authors conceive of human creation, as I will argue. Bajohr and Hayles also claim that electronic texts are more processual than printed texts. They are performative in nature (the code functions as a first text and the output as a second text, with much interplay between them). Therefore, a text produced by an LLM is not a pure imitation of a human text. The code *acts*; it performs meaning even without having any Platonic ideas in its “mind.” Derrida claims that we humans also do not have Platonic meanings in our minds. Of course, this is not to say that we are identical to machines or to algorithmic structures, but the resemblance may be greater than we wish to admit.

Let us consider in further detail the idea that purely human writing is also “representation as repetition in a different mode.” Authors such as Barthes, Foucault, and Derrida claim that the process of writing involves both conscious and unconscious repetition. It is determined by a human mind, a person, defined as the author, but also by “*langage*”—by a system of grammar, by words and their possible meanings, by all previous literary works, and by the contexts in which they are created and received. Here I refer to Barthes’s concept of *langage*, which has its roots in Saussure’s distinction between *langage*, *parole*, and *langue*. The concept of the death of the author underlines the role of *langage* and context in creation. In *Writing Degree Zero *(1970), Barthes claims that the figure of the author is merely a metaphysical myth; in reality, he is simply a product of the “intertext,” the web of all of the idioms and texts that have ever been written, which are interconnected because they refer to each other and whose meaning is defined according to the contexts in which they appear, which entails that they have no Platonic meaning in and of themselves. The author absorbs this intertext and, partly unconsciously, recreates it in a new text. However, this new text is a repetition of other idioms, intertextual references, and quotations, and thus, while it involves a repetition of the same, it also contains new elements as it reemerges in new contexts. Pure originality is impossible, as Barthes argues in *Death of the Author* (1977)*.* Machine-generated texts also emerge from the infinite web of meanings they receive as an input, but as they are also outputs, they engage with infinite chains of new meanings, through which they can be interpreted. LLMs, like the author, also “receive” previously created texts, absorbing them without the direct involvement of consciousness, of a living present. As a result, meaning is separate from consciousness, the soul, or the living present for Derrida, precisely because Derrida was inspired by Barthes and structuralist linguistics. The notion that meaning is created in the human mind alone is therefore false, grounded in metaphysical and Christian presuppositions that view humans as having been created in the image of God. This kind of presupposition establishes a hierarchy between different kinds of entities: animals, things, robots. As David J. Gunkel suggests, the relationship between humans and robots, or humans and machinal mechanisms such as algorithms, is often articulated through metaphors that have their roots in ancient Rome and are related to slavery. But is it at all acceptable to continue to use concepts of this sort, given the problematic historical relations they describe? Gunkel claims that “the extension of the seemingly paradigmatic master–slave relationship to robots, AI systems, and other things is culturally specific and distinctly Western” (2023: 147). For Gunkel, this dynamic poses a danger to both those who position themselves as masters and those who are positioned as slaves, as was the case in colonial slavery. This hierarchy is rooted, in Gunkel’s view, in an anthropomorphic projection.

We can conclude with Derrida that it does not matter whether meaning is created or received by a human mind, an animal, a thing or robot, or an algorithm. Derrida redefines textuality as any kind of transfer of meaning, including the transfer of vibrations, the transfer of seeds by the wind, and the regard of an animal (1982: 309). His definition of meaning is therefore very broad. As a consequence, his definition of “text” is likewise very broad. Derrida writes that every chain of meaning already constitutes a text, and thus he allows for the possibility that animals, nature itself, and machines may be capable of writing autonomous texts with meaning. In the seminar *La vie la mort*, he describes genetic codes as texts, even referring to the “genetic text” (Derrida, 2020: 92). This would seem to be very close to computer scientist Gregory Chaitin’s claim that the entire universe can be considered computer-like (Chaitin, 2007). Derrida provides what would seem to be a similar version of this claim. The very structure of life can thus be understood in Derrida as writing its own meaningful text. As the example of Rousseau’s autobiography shows, human creation—even in the sense grounded in Christian metaphysics—is always already replete with machine-like tendencies. From this perspective, the question whether generative texts are authentic texts is deconstructed.

According to authors like Bajohr, LLMs involve two kinds of text: the code that generates texts or images, which serves as a primary text, and the text that is generated by that code, which serves as a secondary text. The question I have been examining here is whether the generated text (the output) is on a par with human-created literature in terms of its standing as literature. The answer I wish to defend is the following: because the generated text internally entails the secret, the incalculable element, the incomputable, it has value as literature. This of course presupposes that, with Derrida, we conceive of literature precisely in terms of the secret.

A similar position is also defended by Luciana Parisi in *Instrumental Reason, Algorithmic Capitalism, and the Incomputable* (2015) and *Critical Computation: Digital Automata and General Artificial Thinking* (2019)*.* In the latter text, Parisi (2019: 91) claims that there has been a paradigm shift from deductive to non-logical reasoning in machine learning. Deductive reasoning is associated with the Enlightenment and Western rationality, which privileges calculability and seeks to eradicate the ungraspable and the secret from reasoning. Parisi claims that contemporary machine learning relies on abduction, induction, and interactive, adaptive learning algorithms. This is connected to the fact that there is a mutual dependence between data, software, hardware, codes, and algorithms, which has led to a shift from the application of deductive rules to a small set of data towards the “inductive retrieval and recombination of infinite data volumes” (Parisi, 2019: 92). As Parisi observes,

Machine learning is thus the inverse of programming: the question is not to deduce the output from a given algorithm, but rather to find the algorithm that produces this output (Domingos, 2015: 7). Algorithms must then search for data to solve a query. The more data is available the more learning there can be. (Parisi, 2019: 92)

Algorithms work with infinite data sets, thus corresponding to Barthes’s concept of intertextuality. Derrida claims that every text is already an interactive, self-evolving (based on unconscious mechanisms), performative intertext that is marked by iterability. The rules of this interaction cannot be determined in advance; they evolve precisely as their creation unfolds. Thus Derrida describes iterability as leading to the dissemination (the infinite repetition) of meaning, while underlining the unconscious mechanisms at play in creation. Parisi argues that “[a]s rule-obeying behaviours become substituted by the performativity of machinic functions (i.e. what x or y do and do not do, and what they stand for), the indeterminacy of learning outcomes has also become central to the epistemological critique of the end of reason” (Parisi, 2019: 94). She therefore claims that there is a new role for machine learning, one that is based on experimental axiomatics and that has indeterminacy at its core. Error and failure are a part of automated reasoning, which relies on abductive and inductive methods rather than straightforward deduction. The algorithms in question can learn incomplete information and make predictions, without the need for specified predicates. In machine learning mechanisms, abductive reasoning leads to the elaboration of hypotheses and involves working with incomplete information. The model does not simply predict outcomes from a given set of data but actively interacts with incomplete information, forming new hypotheses that lead to new rules and axioms. Abductive reasoning allows it to go beyond logically restricted inferential mechanisms. This is in direct tension with an understanding of AI technologies as “stochastic parrots.” A similar position is defended by Konstantine Arkoudas (2023), who claims that LLMs can produce original outcomes, even though he does not think they will replace human software engineers any time soon. As he argues, “ChatGPT is much more than a stochastic parrot. It can generate novel propositional content and respond to arbitrary questions and scenarios coherently and informatively, and do so in ways that are often strikingly creative” (2023: 4).

Parisi claims that the critique of instrumental rationality should be reformulated insofar as the incomputable cannot simply be opposed to reason (Parisi, 2015). On her view, this is because both digitality and philosophical thought are based on indeterminacy. To support her claim, she refers to Chaitin, who discovered the incomputable number Omega in the field of computer information theory. Omega is characterized by two things: it is possible to define it, but it is not computable. It involves “dynamic processing of infinities in which results are not contained in the logical premises of the system” (Parisi, 2015: 127). We have already touched on the infinitization of contexts in the case of generative texts and machine learning; as Chaitin notes, however, information theory brings to light a second type of infinitization. Parisi claims that directing our attention to the incomputable in information theory problematizes not only technical rationalization but the instrumentalization of reason: “Instead, the limits of automation—that is the incomputable—have become the starting point of a dynamism internal to computation, which exceeds the plan for techno capital’s instrumentalization of reason” (Parisi, 2015: 134). The algorithmic sequences become longer than the instructions, ultimately taking over the set of predefined rules. Parisi thus argues that Chaitin rejects the view of computation, which portrays it as chaotic and random, as an error of calculation.

This can also be viewed in terms of machine learning mechanisms’ resistance to technocapitalism, which echoes Derrida’s description of literature as such, no matter its origin, as a resistance to calculation. Literature is thus defined by the secret within it (see Derrida, 2006: 67–69; Derrida, 2003), defined as resistance to computation, to calculation. In this context, the secret can be identified with incomputability in the field of information theory. As Parisi highlights, according to Chaitin the presence of the incomputable, or the presence of infinite varieties, neither endangers nor annuls computation: “In other words, randomness (or the infinite varieties of infinities) is not simply outside the realm of computation, but has more radically become its absolute condition” (Parisi, 2015: 134). On Parisi’s view, Omega is partially intelligible, and thus it is both intelligible (possible to define) and unintelligible (incomputable). Derrida’s concept of iterability implies the same conclusion within the dynamics of iterability. Iterability is also contradictory in this sense: the iterable is both graspable and ungraspable. Chaitin’s theory refines the internal mechanisms of these dynamics in the context of information theory. Derrida also claims that this paradox is the very condition of iterability. He does not view the secret as excluding repeatability or codability. In the context of information theory, the secret can be understood as randomness, as something that is not understandable by either human minds or machinal algorithms. At the same time, the secret is created in the infinite contexts in which the creation and the interpretation of generated texts take place (*ce qui arrive*, *événement*), involving intertextuality. In short, the secret and codability condition each other.

Therefore, generative literature does not threaten human autonomy and, like human thinking, is based on iterability. The principle of iterability, according to Derrida, includes the secret and non-iterability. Here, however, Derrida does not claim that consciousness (or indeed any other specific origin, such as a body, a brain, a cerebral structure, or a Godlike figure) is needed for creation to take place as an event. He deconstructs these categories and redefines creation such that it can be applied to any contemporary computational creation and such that its creative character can be acknowledged. Because he holds that all creations are always supplemented by something exterior to human consciousness, he claims that any search for the true origin of an artwork must be in vain. There is no true origin of the artwork. The absolutely identical repetition of purely “human” elements by a machine is a myth, as we have seen. The question of autonomy in computational creation should instead be formulated in terms of interactivity and adaptability, which do not imply freedom or intentionality and which move us away from the paradigm of mimesis. This also corresponds to Derrida’s critique of mimesis and intentionality (Russo, 2022; Floridi, 2013).

Once we acknowledge that computational creativity is equivalent to human creation in terms of non-authenticity and authenticity, it becomes clear that this distinction is no longer tenable. As a result, the door is open to imagining remedial uses of LLMs. One possible use that is growing in popularity is automatic translation. Stiegler only acknowledges the negative effects of automatic translation and generative literature, such as the destruction of their milieu, but they have positive aspects as well (2017b: 52). For example, automatic translation provides those who speak so-called “minor” languages with better access to texts that would otherwise not be translated into their language.^7^ It also enables authors who do not speak dominant (or colonizing) languages to write in any such language and to join an (academic, artistic) discourse that would otherwise be closed to them. Another possible use of generative literature is the generation of works in languages that are viewed as mere dialects and are in danger of disappearing. This is the case, for example, in parts of Brazil and northern Turkey, where the production of such literature has been difficult due to a lack of funding in the cultural sector and a lack of incentive to grant certain languages official status.^8^ LLMs open the door to the easy and accessible diffusion of these languages and could be used to prevent their disappearance. Given their present stage of development, however, their ability to do so presupposes the existence of a digitalized archive from which they can learn. The biggest issue in this regard is the fact that LLMs are controlled by capitalist systems and that their production is centralized. Neural networks of this kind are trained on Reddit and Twitter, which may be problematic insofar as their “first language” is English (see Floridi & Chiriatti, 2020). Nevertheless, as shown by the example of Lisa Gennart (*Výsledky vzniku*)—a machine learning program (GPT-2) that was trained on the digital archive of Slovak literature—such a use is indeed possible. Rather than rejecting these mechanisms categorically, what is needed is a constructive critique of their use. Such a critique would point to a reality that differs fundamentally from the reality of capitalism. As Parisi claims, imagining the incomputable offers a way out of capitalist, instrumental uses of reason. This is possible precisely thanks to the very tools that Stiegler interpreted as associated with the “destruction of public power” (2017b: 41). Indeed, AI is a *pharmakon* in the true, dual sense of the word: something that is neither good nor bad per se but that becomes good or bad depending on its use, like the fire of Prometheus. Stiegler claims that the digitalization of traces (and of knowledge in general) leads to the destruction of desire and of the libidinal economy. But is it not conceivable that the diffusion of “minor” languages could encourage new forms of desire that can be experienced outside of a Western-oriented, Eurocentric capitalistic cultural space? Theoretically, this could also contribute to the generation of new social forms, including forms of resistance to oppressive governments, precisely because they can be shared online (for example the diffusion of texts in minor languages or dialects that we have seen in countries such as Brazil and Turkey). Rather than necessarily leading to the disintegration of psychic and collective retentions and protentions (memories and future projections), as Stiegler claimed, automatic creation may in fact give rise to new collective forms of consciousness and affects.

## Footnotes

1 This work was supported by John Templeton Foundation project Persons of Responsibility: Human, Animal, Artificial, Divine.

2 Zuzana Husárová claims that the author of the book is Liza. Husárová and her husband, who co-created Liza, consider themselves authors of the author (Husárová, 2022: 77).

3 In October 2018, the first scientific book was translated by DeepL and released in French (Goodfellow et al., 2018. The translation was directed by Goodfellow et al. (2016).

4 The theoretical framework proposed by Frederica Russo explains what is at stake in machinal creativity, defined as *poiêsis*. Her account explicitly redefines the Greek (and, I would say, the Heideggerian) account of *poiêsis* as creative production. *Poiêsis* is defined by Russo as cooperation between human epistemic agents and instruments (including algorithms and machine learning mechanisms), the latter of which are artificial agents and have a poietic character. See Russo (2022: 186).

5 Derrida defines an “event” as something that occurs in the opening of possibilities—“*ce qui arrive*.” In this paper, I treat artworks as falling under this category (Derrida, 2007: 441–461).

6 On Derrida and technology, technique, and virtuality, see Warminski (2009) and Brown (2014).

7 Here, I allude to Gilles Deleuze’s and Félix Guattari’s concept of minor literature (1983). Deleuze and Guattari include Kafka’s works under the category of “minor” literature. This view is highly problematic to the extent that Kafka remains a mainstream, canonical author in the context of Slovak and Czech literature studies. He has also been appropriated within German literary theory as a German author, although he was Czech and wrote in German, which only reflects how deeply Western European (in particular French and German) appropriative attitudes toward Slavic literature and languages continue to run.

8 For the application of Derrida’s thought to the post-colonial context of Brazil, see Rocha (2020) and Mariani (2003).

## Declarations

I give my consent for publication of this paper by Philosophy and Technology. The paper is not under review in any other journal and it was not published yet.
